# Is time an embodied property of concepts?

**DOI:** 10.1371/journal.pone.0290997

**Published:** 2023-09-05

**Authors:** Charles P. Davis, Eiling Yee

**Affiliations:** 1 Department of Psychology & Neuroscience, Duke University, Durham, North Carolina, United States of America; 2 CT Institute for the Brain and Cognitive Sciences, University of Connecticut, Storrs, Connecticut, United States of America; 3 Department of Psychological Sciences, University of Connecticut, Storrs, Connecticut, United States of America; Northumbria University, UNITED KINGDOM

## Abstract

A *haircut* usually lasts under an hour. But how long does it take to recognize that something is an *instance* of a haircut? And is this “time-to-perceive” a part of the representation of concepts like *haircut*? Across three experiments testing lexical decision, word recognition, and semantic decision, we show that the amount of time people say it takes to perceive a concept in the world (e.g., *haircut*, *dandelion*, or *merit*) predicts how long it takes for them to respond to a word referring to that thing, over and above the effects of other lexical-semantic variables (e.g., word frequency, concreteness) and other variables related to conceptual complexity (e.g., how confusable a concept is with other, similar concepts, or the diversity of the contexts in which a concept appears). These results suggest that our experience of how long it takes to recognize an instance of a concept can become a part of its representation, and that we simulate this information when reading words. Consequently, we suggest that time may be an embodied property of concepts.

## Introduction

If asked how long a haircut takes, a common answer would probably be, “Generally under an hour.” But if asked how long it takes to recognize an *instance* of a haircut, a more likely response would be, “Probably just a few moments.” That is, experiencing an entire haircut unfold is different than perceiving the elements that make up a haircut (e.g., a barber, customer, and sufficient evidence that cutting hair is in order). And even though we might rarely, if ever, consciously think about how long it takes to perceive something, as this example shows, we do have knowledge about it. Here we consider whether how long it takes to perceive something is part of what we know about that thing—and more specifically, whether we implicitly re-enact that experience such that the longer it takes for us to perceive something in the world, the longer it takes to play out in our minds.

Our conceptual knowledge (our knowledge of what, e.g., a *haircut* or *merit* is) is the lens through which we perceive the world. But what type of information does conceptual knowledge include, and how it is represented in the mind? Contemporary investigations have largely focused on how sensory and motor experiences shape our concepts (e.g., [1–8]), although some researchers have begun considering the role of other types of experience, such as emotional [9], interoceptive (i.e., sensations within the body [10]), and distributional information from language (for review, see [11]), suggesting that each of these types of information is activated when we process the meaning of concepts like *merit*, *haircut*, or *grapefruit*. A type of experience that has received less attention with respect to its contribution to conceptual knowledge, however, is temporal unfolding, broadly construed (but see, e.g., [12–15])—and in particular, how much time it takes to *perceive* an instance of a concept (i.e., time to accumulate the information needed to apprehend an instance of it) has been largely ignored.

This is not to say that time has been entirely disregarded as a component of conceptual knowledge. In fact, time has recently been suggested as a feature of concepts [16–19], but these accounts have focused on the degree to which concepts are *associated with* time (e.g., concepts like *event*, *clock*, and *race* may be associated with time), rather than considering which components of temporal experience may modulate conceptual knowledge (but cf. [20]). Such components could include (among others) *duration* (e.g., of a generalized event concept like *haircut*), *sequencing* (i.e., the order in which events unfold; when getting a *haircut* one sits in the chair before the barber begins cutting), or the time that it takes to *perceive* an instance of a concept (e.g., the time it takes to apprehend evidence of hair cutting). Here, we focus on this latter component.

Although “time-to-perceive” may seem an unlikely property of concepts, according to experience-based (e.g., embodied) theories, any systematic component of our experiences with concepts should become part of conceptual knowledge [21]. And the time that it takes to perceive an instance of a concept is just as much a part of the experience that we have with a concept as other, more-studied experiential properties. In fact, research on how people comprehend generalized *events* via language suggests that when processing sentences describing events, people simulate a related aspect of time—the time it would take for an event to unfold. For example, it takes longer to read sentences describing ongoing events (e.g., “we were approaching the summit”) compared to punctive events (e.g., “we reached the summit”), and these reading times are correlated with ratings of how long people think these events would take to unfold [12]. This suggests that our knowledge about the amount of time an event takes is generalized from our experiences with that event and stored in long-term memory [22–25]. However, that knowledge is imprecise—how we think about time is influenced and distorted by several factors, such as physical space and the number of sub-events in an event representation, e.g., [13, 14, 26–29]). And consistent with the idea that retrieving knowledge about how long an event takes involves simulating its duration, there is electrophysiological evidence that making duration judgements about events recruits superior parietal brain regions that are involved in temporal processing [30].

But what about the individual concepts that make up events? Although it seems nonsensical to ask how long it takes for many individual concepts to *unfold* (e.g., it seems nonsensical to consider how much time it takes to experience the unfolding of a *dandelion* or a *grapefruit* in the sense of experiencing an event unfolding), we *can* ask how long it takes to *perceive* instances of individual concepts. For instance, while the parts that make up a *grapefruit* can be perceived together in a temporally bound experience, the elements that comprise *tradition* are more likely to be spread across time (e.g., the routines that unfold on Christmas morning may be spread over the course of minutes or hours) such that apprehending an instance of *tradition* takes longer. In other words, whereas a *grapefruit* can be readily perceived in a single, temporally circumscribed “snapshot,” apprehending *tradition* requires detecting and perceiving multiple, temporally dispersed elements. Thus, how much time it takes to accumulate the information needed to perceive an instance of a concept is something that is part of our experience with concepts, and it varies across them (for further discussion, see [20]).

This brings us to the question at hand: Does this experience of how long it tends to take to perceive an instance of a concept become part of its long-term representation? And if so, when conceiving of something, do people implicitly simulate the amount of time that it takes to recognize an instance of it? We hypothesized that if time is “embodied” in the sense that we simulate the amount of time that it takes to perceive an instance of something when we think about that thing, then things that take more time to perceive should take more time to conceive of. To test this, we collected people’s ratings of the degree to which a concept requires a relatively long or short period of time to perceive, hypothesizing that these ratings should predict how long it takes to think about that concept (as measured by response times when that concept is presented as a word).

Because we anticipated that time-to-perceive would covary with other lexical and semantic variables, we controlled for these in our analysis as follows. First, we asked people to rate how much *space* a concept would require to perceive (i.e., we asked about the degree to which its elements are spread over space). If an effect of time-to-perceive is actually about how much physical space the elements to be integrated are spread across, then the effect should be accounted for by these space ratings. Notably, although we speculate that space-to-perceive *is* a feature of concepts [20], unlike for time-to-perceive, there is no obvious link between simulating more space to perceive and longer reaction times, beyond that accounted for by any overlap between *space*- and *time*-*to*-*perceive*. Thus, we do not expect response times to be predicted by space ratings.

We also collected ratings on “confusability,” i.e., how easy it is to confuse a given thing with other, similar things. We collected these ratings because pilot work suggested that some participants conflated the time it would take to integrate the information necessary to perceive a concept with the time it would take to distinguish something from a similar concept (e.g., although it may not take long to simply perceive the properties that make up a *banjo*, it may take longer to determine that it *is* a *banjo*, and not a highly similar object such as a *mandolin*), yet we wanted to keep these constructs separate. In addition, we controlled for several other relevant lexical-semantic variables (word length, word frequency, concreteness, and age of acquisition) which might impact on people’s reports of how long it takes to perceive different concepts, and which are known to correlate with reaction times.

We also conducted supplemental analyses controlling for two less commonly used measures: semantic diversity as defined in [31] (which quantifies the dissimilarity of all the contexts in which a word appears in a large text corpus and thus is related to word ambiguity) and visual perceptual strength (a measure related to imageability [32]); these additional variables were included in exploratory analyses because one might imagine that time-to-perceive ratings are affected by the semantic ambiguity of the word being rated (and therefore effects of time-to-perceive would be accounted for by semantic diversity) or by how difficult it is to visualize the concept (and therefore effects of time-to-perceive would be accounted for by imageability). Including all of these controls is necessary to focus our investigation on whether there is a role of *time*-*to*-*perceive* in concept processing and rule out competing explanations.

We tested our predictions in three experiments utilizing separate datasets from three existing mega-studies which each collected response times on a distinct task: (1) lexical decision (i.e., is this a real word in English? [33]), (2) word recognition (i.e., do you know this English word? This task is similar to lexical decision, but differs in that participants are not speeded, and are only asked to indicate words which they, personally, know in English [34]), and (3) semantic decision (i.e., is this word concrete or abstract? [35]).

We included three separate tasks for two reasons. First, the tasks differ in the depth of processing required: lexical decision is a more “superficial” task in that it can be performed based on overall familiarity with the letter-string (e.g., “is this letter-string more likely to be a word or a non-word?”), whereas the word recognition task is somewhat deeper in that participants were instructed to respond positively only if the word was part of their own vocabulary (that is, they were asked to avoid guessing on words they think could be real words [34]), and the semantic decision task is deeper still, as it requires accessing (at least some aspect of) the word’s meaning. Thus, given that the amount and type of information available about a word can be affected by the task (e.g., is sensorimotor simulation necessary, or is a linguistic shortcut sufficient? for review, see [36, 37]), if our results differ between tasks, this could reveal the level(s) at which time-to-perceive information becomes available. For instance, observing an effect only in semantic decision would suggest that *time*-*to*-*perceive* information becomes available only during relatively deep processing of word meaning, whereas if we also observe an effect in lexical decision, this would suggest more routine activation of *time*-*to*-*perceive* upon reading a word.

Second, including three separate tasks—each conducted on a separate group of participants—offers a test of the robustness of the effects: observing consistent and statistically reliable effects of *time*-*to*-*perceive* across tasks would provide stronger support for the hypothesis that we encode the time it takes to perceive concepts, and that we reactivate that information during concept processing.

## Methods

### Materials

We selected 650 relatively high-frequency noun-dominant English words (as verified by part-of-speech tags in Brysbaert et al., [38]) that cover a range of concreteness according to Brysbaert et al.’s [38] concreteness norms and are present in Pexman et al.’s [35] Calgary Semantic Decision Project. For these words, we collected ratings on the *time* and *space* it would take to perceive the concept to which each word refers, as well as on that concept’s *confusability* (i.e., how difficult it is to distinguish that thing from something similar). In addition, because our goal was to assess whether temporal properties of concepts contribute to word processing over and above properties that are already well-known to affect lexical-semantic processing, for all of our items, we also calculated word length and obtained measures of word frequency (log-transformed frequency from a subtitle corpus; [39]), and concreteness [38]. Although we collected data on 650 words, after data collection, we realized that controlling for age of acquisition—a measure of word familiarity—was critical, so we also obtained age of acquisition measures, which were available for 634 of the words [40]. Thus, our analyses center on the 634 words for which age of acquisition ratings were available. Two less commonly used measures, semantic diversity [31], which is a measure of conceptual ambiguity (i.e., it assesses the dissimilarity of all the contexts in which a word appears) and visual perceptual strength [32], a proxy for imageability, were also included in supplemental analyses.

### Data collection

Data were collected between April 2020 and February 2021. The stimuli were divided into two lists, each with 325 words. The two lists were balanced for word length, word frequency, and concreteness using the R package *LexOPS* [41]. The lists were administered to 240 undergraduate students; each participant saw just one list, and rated the words in that list on one dimension (time, space, or confusability). All responses were completely anonymous. Participants were excluded for either leaving large portions of the survey unanswered (*n* = 15) or for indicating that they did not speak English as a first language (*n* = 5), leaving *N* = 220 (*M*_age_ = 19, 64 males, 152 females, 3 preferred not to say, 1 no response). Thus, each word was rated on each dimension by 33–39 participants. The following instructions were used to orient participants to the *time*-*to*-*perceive* dimension, where participants rated each item on a 7-point scale from 1 (*very little time*) to 7 (*a lot of time*).

*You will be asked how long it takes to perceive different things*.

*For example*, *you typically do not need much time to accumulate the information needed to perceive a bowl—e*.*g*., *its shape and size can be immediately observable to the senses*. *But for other things*, *like tradition*, *it may take longer to accumulate the necessary information—e*.*g*., *it may require perceiving multiple events spread across time*. *Still other things*, *like gamble*, *thinking*, *or galaxy may lie somewhere in between*.

*Make your responses based on how long it would take you accumulate the information necessary to perceive the parts that make up each thing*.

*We are* ***not*** *interested in how easy it is to tell each thing apart from something similar*. *For example*, *even if you think it would be hard to distinguish a banjo from*, *e*.*g*., *a mandolin*, *it does not take long to perceive the parts that make up a banjo*. *A banjo would therefore likely receive a response on the “very little time” end of the scale*.

*We are also* ***not*** *interested in how familiar you are with each thing*. *For example*, *even if you are unfamiliar with mandolins*, *it does not take long to perceive the parts that make up a mandolin*. *A mandolin would therefore likely receive a response on the “very little time” end of the scale*.

*Please tell us how long you think it would take to accumulate the information needed to perceive the following things*. *There are no right answers*, *so simply go with your first instinct*.

Similar instructions were given to elicit space-to-perceive and confusability ratings (see S1 and S2 Appendices). Each survey took about 30 min to complete. Participants provided written informed consent prior to participating, and were compensated with course credit. The procedures were approved by the University of Connecticut Institutional Review Board.

## Results

**Fig 1:**
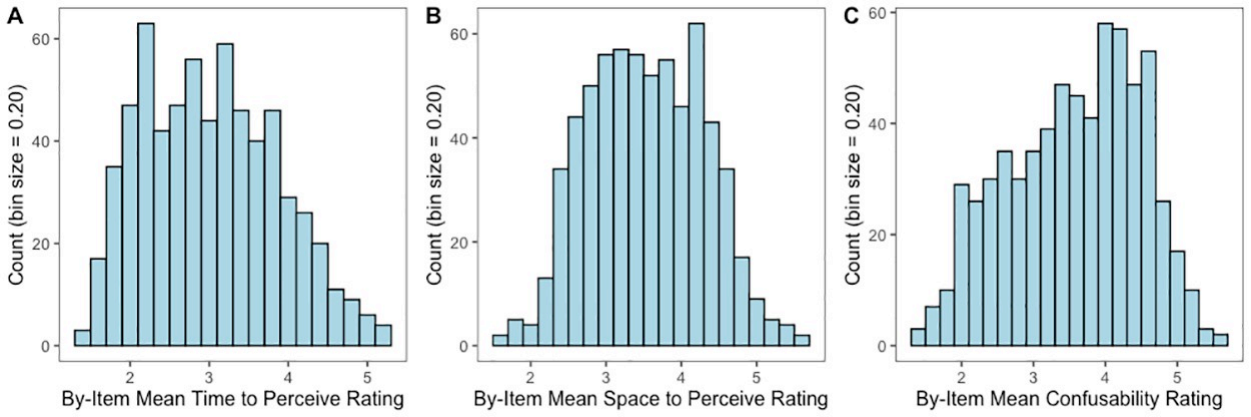
Histograms showing the rating distributions of novel time-to-perceive, space-to-perceive, and confusability variables. Each variable is rated on a 1–7 scale.

Summary statistics for the ratings on time-to-perceive, as well as those for space-to-perceive and confusability are shown in Table 1, and the distributions are shown in Fig 1. Correlations among all of the variables included in our main and supplemental analyses are shown in Table 2.

**Table 1:** Mean, standard deviation, and range for time, space, and confusability ratings.

Word type	Mean	SD	Range
Time	3.01	0.85	1.46–5.30
Space	3.54	0.76	1.67–5.54
Confusability	3.56	0.91	1.39–5.70

**Table 2:** Correlation matrix for all variables.

	Length	Freq	AoA	Conc	Visual	SemD*	Time	Space	Confus
Length	—								
Freq	-.30	—							
AoA	.20	-.47	—						
Conc	.03	-.04	-.40	—					
Visual	.07	.02	-.37	.63	—				
SemD*	-.06	.37	-.06	-.42	-.17	—			
Time	.09	-.27	.72	-.65	-.52	.16	—		
Space	.18	.07	.45	-.69	-.44	.26	.66	—	
Confus	.03	-.27	.68	-.56	-.51	.11	.85	.53	—

*Note*. Freq = subtitles word frequency; AoA = age of acquisition; Conc = concreteness; SemD = semantic diversity; Visual = visual perceptual strength; Confus = confusability.

*only available for 469 items; all others are based on the 634 words for which AoA ratings are available.

Our critical hypothesis was that the temporal characteristics of experience are part of a concept’s representation, and as a consequence, are activated during lexical-semantic processing. Thus, in each of the three datasets (lexical decision, word recognition, and semantic decision) we tested whether time to perceive a concept accounts for significant variance in response times over and above space-to-perceive, confusability, concreteness, and common psycholinguistic variables that are well-known to affect response times: namely word frequency, word length and age of acquisition (and in supplemental analyses, semantic diversity and visual perceptual strength).

Data were analyzed using R statistical programming software [42]. To evaluate our hypothesis, for each of the three datasets we constructed linear regression models in three steps (these steps were identical across the three datasets with one exception, described below). In Step 1, we entered word length, log-transformed word frequency [39], age of acquisition [40], and concreteness [38]. In Step 2, we entered our novel variables controlling for conceptual complexity, namely, space-to-perceive and confusability. In Step 3, we entered time-to-perceive, the critical variable for testing our hypotheses. The general analytical approach is motivated by methods in Pexman et al. [35] as well as those in Juhasz and Yap [43] and Tillotson et al. [44] for evaluating the effects of a novel semantic variable on lexical-semantic decision times.

In our first experiment, we analyzed lexical decision data from the English Lexicon Project (ELP [33]). Because 24 of our words were not available in the ELP database, our ELP models contain 610 words. In our second experiment, we implemented an identical model to evaluate our predictions in the context of word recognition data from the English Crowdsourcing Project (ECP [34]). And in our third experiment we implemented the same model to evaluate our predictions in the context of semantic decision data from the Calgary Semantic Decision Project (SDP [35]). For the semantic decision task, because the task is an abstract-or-concrete decision, task difficulty is necessarily influenced by each word’s proximity to the middle of the concreteness scale. Thus, for these models only, we also included a “distance” measure, which is simply the midpoint of the concreteness scale (3 on a 1–5 scale) subtracted from the observed concreteness rating (e.g., for *dandelion*, 5, 5–3 = 2). The absolute value was taken as a measure of “distance,” and so higher values (up to 2) reflect easier decisions. (Although the addition of this distance measure did not change the pattern of results, it clearly accounted for significant variance in response times, and some effects became stronger; we thank an anonymous reviewer for suggesting it.).

All ratings were averaged by word, and analyses were performed at the word level. For all models, we report the unstandardized estimates as effect sizes, along with their standard errors and associated *t*- and *p*-values. Successive models (i.e., Step 2 vs. Step 1, Step 3 vs. Step 2) were compared using ANOVA. Here, we report *F*-values and *p*-values (*p* < .05 was the threshold at which predictors were considered statistically significant, and at which successive models were considered to be a significantly better fit to the data). Our data and R analysis scripts are publicly available (https://osf.io/q2gdt/).

In each experiment, a direct comparison of Model 2 and Model 3 indicated that Model 3, which included *time*-*to*-*perceive*, was a better fit to the data than was Model 2, which included all lexical-semantic control variables in addition to our novel conceptual complexity control variables (space-to-perceive and confusability). Detailed model results are presented in Table 3. In fact, once time-to-perceive was entered into the analysis (across experiments, in Model 3), only time-to-perceive was a reliable predictor of response latencies among the conceptual complexity variables; there was no effect of the amount of space required to perceive a concept in any of the three experiments, and a concept’s confusability with other, similar concepts was also non-significant (although as we come to next, the effect of confusability in Model 3 is likely an underestimate).

**Table 3:** Model results for response times in word recognition, lexical decision, and semantic decision tasks.

	Lexical Decision (ELP; Balota et al. [33]) *N*_words_ = 610				Word Recognition (ECP; Mandera et al. [34]) *N*_words_ = 634				Semantic Decision (SDP; Pexman et al. [35]) *N*_words_ = 632			
**Model 1**	**est**	***SE***	***t***	***p***	**est**	***SE***	***t***	***p***	**est**	***SE***	***t***	***p***
Length	13.67	1.67	8.18	**< .001**	10.78	2.03	5.32	**< .001**	7.84	2.36	3.33	**< .001**
Frequency	-24.68	2.74	-9.00	**< .001**	-32.23	3.28	-9.82	**< .001**	-8.41	3.83	-2.19	**.029**
AoA	12.54	1.56	8.05	**< .001**	14.16	1.88	7.51	**< .001**	12.18	2.24	5.44	**< .001**
Concreteness	7.02	3.32	2.12	.**03**	14.54	4.05	3.59	**< .001**	-11.64	5.51	-2.12	**.035**
Distance* (to concreteness midpoint)	—	—	—	—	—	—	—	**—**	-119.85	13.86	-8.65	**< .001**
**Total variance explained**	***R***^***2***^ ***=* .*45***				***R***^***2***^ ***=* .*40***				***R***^***2***^ ***=* .*37***			
**Model 2**	**est**	***SE***	***t***	***p***	**est**	***SE***	***t***	***p***	**est**	***SE***	***t***	***p***
Length	13.10	1.76	7.43	**< .001**	11.66	2.10	5.56	**< .001**	8.38	2.50	3.34	**< .001**
Frequency	-26.19	2.89	-9.06	**< .001**	-31.95	3.39	-9.41	**< .001**	-7.74	4.07	-1.90	.06
AoA	9.06	1.88	4.81	**< .001**	6.66	2.21	3.01	**.003**	10.92	2.65	4.12	**< .001**
Concreteness	15.74	4.32	3.65	**< .001**	28.46	5.16	5.52	**< .001**	-10.52	6.54	-1.61	.11
Distance* (to concreteness midpoint)	—	—	—	—	—	—	—	**—**	-118.17	14.12	-8.37	**< .001**
Confusability	12.24	5.27	2.32	**.021**	36.44	7.75	5.82	**< .001**	8.20	7.51	1.09	.28
Space	12.81	6.49	1.98	**.049**	8.09	6.26	1.04	.30	-2.27	9.31	-0.24	.81
**Total variance explained**	***R***^***2***^ ***=* .*46 F(2*, *603) = 5*.*60*, *p =* .*004***				***R***^***2***^ ***=* .*44 F(2*, *627) = 19*.*14*, *p <* .*001***				***R***^***2***^ ***=* .*37 F(2*, *624) = 0*.*60*, *p =* .*55***			
**Model 3**	**est**	***SE***	***t***	***p***	**est**	***SE***	***t***	***p***	**est**	***SE***	***t***	***p***
Length	13.55	1.74	7.77	**< .001**	12.10	2.06	5.86	**< .001**	8.79	2.48	3.54	**< .001**
Frequency	-24.50	2.88	-8.50	**< .001**	-29.98	3.37	-8.90	**< .001**	-5.97	4.06	-1.47	.14
AoA	6.80	1.94	3.52	**< .001**	3.69	2.27	1.62	.10	8.18	2.74	2.98	**.003**
Concreteness	19.64	4.36	4.50	**< .001**	33.68	5.20	6.47	**< .001**	-5.25	6.65	-0.79	.43
Distance* (to concreteness midpoint)	—	—	—	—	—	—	—	**—**	-120.95	14.02	-8.62	**< .001**
Confusability+	-4.68	6.59	-0.71	.48	14.46	7.79	1.86	.06	-11.92	9.42	-1.26	.21
Space	4.02	6.74	0.60	.55	-3.19	8.01	-0.40	.69	-12.63	9.70	-1.30	.19
Time	36.08	8.63	4.18	**< .001**	46.99	10.18	4.62	**< .001**	42.60	12.25	3.48	**< .001**
**Total variance explained**	***R***^***2***^ ***=* .*47 F(1*, *602) = 17*.*48*, *p <* .*001***				***R***^***2***^ ***=* .*45 F(1*, *626) = 21*.*30*, *p <* .*001***				***R***^***2***^ ***=* .*38 F(1*, *623) = 12*.*09*, *p <* .*001***			

*Note*. ECP = English Crowdsourcing Project; ELP = English Lexicon Project; SDP = Calgary Semantic Decision Project; AoA = Age of Acquisition. The ANOVA summary in the final row for Models 2 and 3 shows the results of an ANOVA comparing Model 2 to Model 1, and Model 3 to Model 2, respectively.

* Distance was computed as the difference between an item’s measured concreteness value and the midpoint of the concreteness scale. This was included (only in the semantic decision models) because judging whether a word is concrete or abstract is presumably most difficult near the midpoint of the scale (i.e., in boundary cases), and so accounting for distance allows us to better capture variation in semantic decision RTs. (Without it, the patterns were the same, and time-to-perceive was still a reliable predictor of response times, but the total variance explained by the semantic decision models was substantially lower: *R*^*2*^ = ~.30.)

^+^ See text for discussion of the contribution of confusability to this model.

As shown in Table 2, however, correlations among some of our predictor variables were quite high, which could lead to collinearity concerns. In fact, the correlation between confusability and time-to-perceive was especially high (*r* = .85), and when we computed the variance inflation factor (VIF) for each of the predictor variables in Model 3, we observed that for the critical time-to-perceive variable and for confusability, the VIFs approach levels at which it becomes difficult to determine which predictor may be explaining the variance in the dependent variables (VIFs of 5.6 and 3.8, respectively; the VIFs for the other variables in Model 3 are < 3, and so are not of substantial concern). It is therefore important to note that our statistical approach (model comparison of simultaneous multiple regressions) is conservative; the multiple regression provides estimates of each predictor variable’s contribution while holding the others constant (i.e., any variance shared between predictors is not attributed to any individual predictor), and the model comparison evaluates the unique contribution of the added predictor(s).

Consequently, we can be confident that time-to-perceive indeed accounts for significant variance in reaction times (i.e., Model 3’s estimate of the contribution of time-to-perceive is conservative—it may be an underestimate). Although confusability is only included in the model as a control, it is worth noting that its contribution is also likely underestimated in Model 3. In particular, confusability’s relatively high VIF in Model 3, coupled with the fact that between Model 2 (in which all VIFs are below 2.5) and Model 3 there is a considerable change in the estimate of its contribution, suggests that the true effect of confusability lies somewhere between the estimates in Model 2 and Model 3.

Overall, the model results demonstrate that time-to-perceive is a reliable and independent predictor of response latencies. Specifically, in each of the three datasets (each using a different task), we found substantial evidence that the time it takes perceive an instance of a concept predicted the amount of time it takes to process a word referring to it, over and above the control variables.

As an additional, exploratory check of whether time-to-perceive has a role in conceptual knowledge that is separate from other variables that may seem likely to covary with it, we also created models that include semantic diversity (i.e., how semantically dissimilar are the contexts in which a word tends to appear [31]) and a proxy for imageability—visual perceptual strength (i.e., ratings on how strongly the concept is experienced by seeing [32]). We report these models separately from the primary analyses above because (1) of their exploratory nature, (2) only 459 of the 650 words in the full dataset were present for all experiments in the semantic diversity dataset (even after converting UK to US spellings), and (3) unlike the other sources of data used in this study, the semantic diversity norms are derived from UK English. The models including semantic diversity reveal that semantic diversity was not a significant predictor of response latencies in any of the three datasets (nor was it strongly correlated with time-to-perceive ratings; Table 2). Furthermore, despite the smaller size of the dataset, time-to-perceive remained a reliable predictor of response latencies in all three datasets. In the models including visual perceptual strength (as a proxy for imageability, instead of controlling for concreteness) this factor was a reliable predictor of RTs for word recognition and lexical decision, but as with the semantic diversity models, adding visual perceptual strength did not change the critical result in any of the models—time-to-perceive remained a reliable predictor in all three datasets. Full model details are provided in in S1 Table (for semantic diversity) and S2 Table (for visual perceptual strength) of the Supplementary Material.

**Fig 2:**
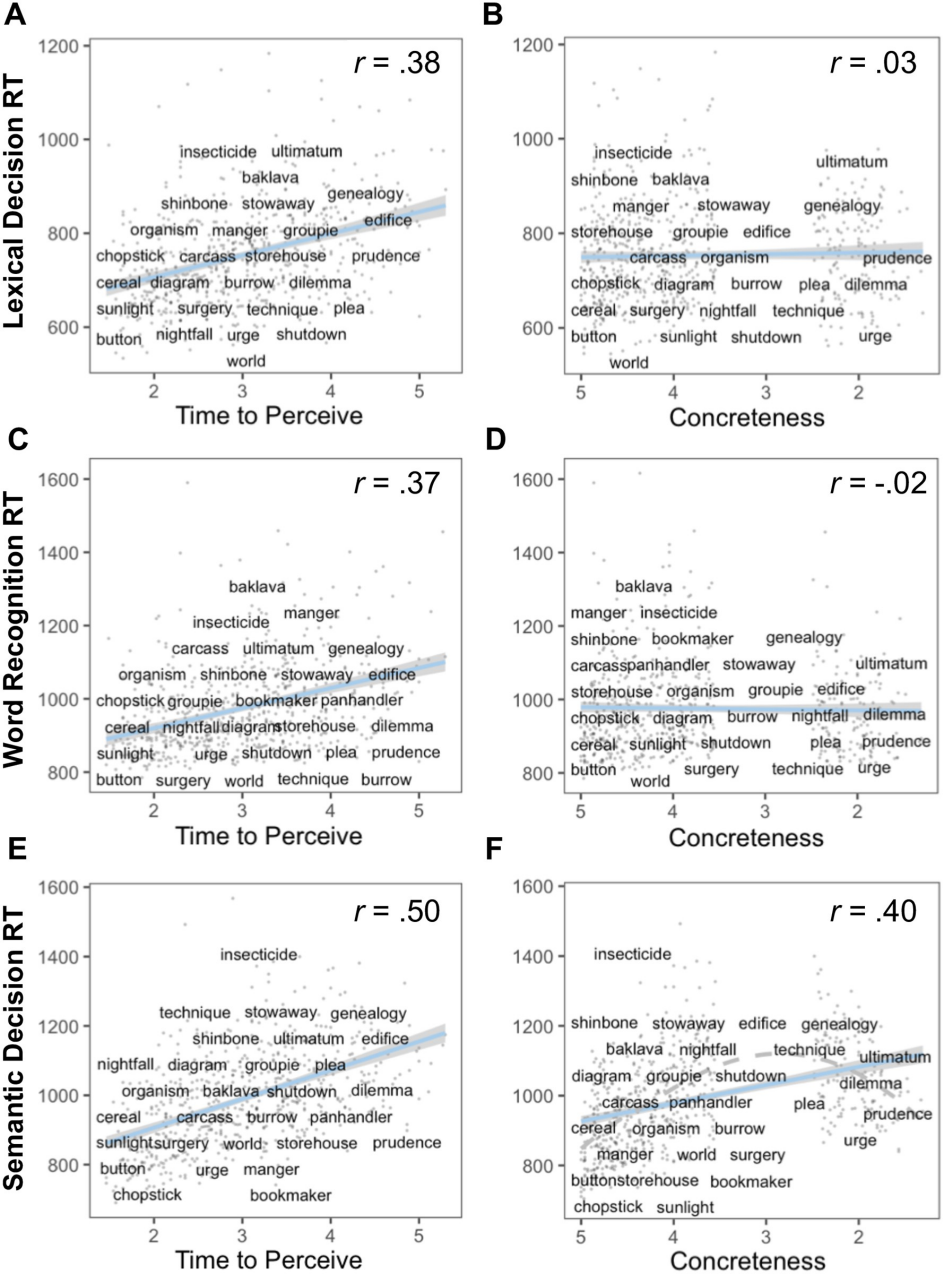
Correlations between time-to-perceive and decision latencies (left panels) and concreteness and decision latencies (right panels; to facilitate comparison with time-to-perceive, we have reversed the concreteness scale [the signs on the *r* values correspond to the reversed scale] so that more abstract items appear to the right side). Plots show the positive relationship between decision latencies and time-to-perceive in each experiment (left panels), and that this relationship is different for concreteness (right panels). For ease of interpretation, we use raw scores on the x-axis in both cases. Note that although effects of concreteness are less evident in these first-order correlations, this does not mean that concreteness has no bearing on decision latencies (as Table 3 indicates, there is an effect of concreteness after accounting for word length, frequency, and age of acquisition); rather, plots illustrate that time-to-perceive and concreteness have disparate effects on decision latencies. The full range of concreteness is not present in the data because we wanted to test the same set of words on all three tasks, and Pexman et al. [35] only included words with concreteness values > 3.5 and < 2.5 for their semantic decision (abstract or concrete?) task. To provide examples of the items, data points corresponding to a random set of words (5% of the total word list) are labeled in each panel. However, all data points appear in each panel (as light gray points). The dashed gray line over panel F shows the inverted U-shaped function originally reported in Pexman et al. [35], which we fit here as a LOESS (locally estimated scatterplot smoothing) curve, showing that decision latencies are indeed slowest in the abstract/concrete boundary cases.

To help visualize the relationship between time-to-perceive and response times, Fig 2 (left panels) illustrates the relationship on each of these tasks. Because one might imagine that time-to-perceive simply tracks concreteness in predicting response times, we also show that the relationship between concreteness ratings and response times on each of these tasks (right panels) differs from that of time-to-perceive. For ease of interpretability, we use raw scores on the x-axis in both cases. Importantly though, although effects of concreteness are not apparent in these first-order correlations, we do not mean to suggest that concreteness does not impact response times—as the model results indicate, it does when word length, frequency and age of acquisition are accounted for. Rather, the correlations are intended to illustrate that the effect of time-to-perceive on response times is dissociable from that of concreteness. Note that in Fig 2f, although the linear fit implies a positive relation between concreteness and semantic decision RTs, the relationship is in fact an inverted U-shape (we plot this as a dotted gray line fitting a LOESS curve), as in Pexman et al. [35]. This is because the semantic decision that participants were asked to make was an abstract/concrete judgment, and this judgment is easier at both extremes of the distribution (i.e., where the correct response is most obvious) than in the middle/boundary cases (this feature of the semantic decision task was the motivation for including the “distance to concreteness midpoint” measure in the models reported in Table 3).

## Discussion

Across three different datasets with three distinct tasks, we found that ratings of how long it takes to *perceive* something in the real world predict how long it takes to process a word referring to it. Critically, the effect of time-to-perceive on processing times for words was observed after accounting for effects of relevant lexical-semantic variables (word frequency, age of acquisition, word length, and concreteness), as well as after accounting for additional control variables, namely confusability (how difficult it is to distinguish one concept from another), and the space required to perceive a concept. Space-to-perceive and confusability were important controls because (1) concepts that take more time to perceive likely also involve more elements or sub-events, and are thus spatially extended, and because (2) concepts that are more confusable tended to be rated as taking more time to perceive—both for relatively concrete things, like *violin* and *viola*, and more abstract concepts like *agreement* and *amendment*.

It is worth considering confusability in more detail because although our conservative analysis approach means that we can be confident that time-to-perceive does impact reaction times (i.e., if anything, the effect of time-to-perceive is *larger* than we report, and the same goes for the effect of confusability), the correlation between confusability and time-to-perceive was quite high—perhaps because until the thing is perceived, it is difficult to distinguish it from other things. We therefore performed a further exploratory check of the effect of time-to-perceive separated from that of confusability by limiting our analysis to 180 words where confusability’s range is limited (2.5–3.5) but time-to-perceive still varies ~1.5–4 (thereby reducing the correlation between the two to *r* = .46). In this analysis, the effect of time-to-perceive remains statistically reliable in all three tasks (full exploratory model results are available on the OSF; https://osf.io/q2gdt/). This suggests that although time-to-perceive correlates with confusability, it nevertheless has a dissociable, independent effect on conceptual processing.

In additional supplemental analyses (S1 and S2 Tables), we also controlled for two other meaning-related measures: semantic diversity and visual perceptual strength. We controlled for semantic diversity (i.e., a measure of how semantically dissimilar are the contexts in which a word appears, which is a proxy for semantic ambiguity) because it seemed plausible that words referring to things that take more time to perceive are more ambiguous than other words (e.g., although we used only noun-dominant items, some items could be more noun-dominant than others, or there could be ambiguity within the noun part-of-speech) and it is this ambiguity that slows responses, perhaps due to competition between meanings, or additional time needed to access more diverse contexts. Even though we were only able to include two-thirds of our items in this analysis due to missing semantic diversity scores for the remaining items, the effect of time-to-perceive remained significant in all three datasets. Furthermore, semantic diversity was not a reliable predictor of response times in any of the datasets. This contrast may be of interest in light of discussion about what mechanism accounts for the finding (described in the introduction) that there is a positive relationship between reading times and duration ratings for *events* described in sentences [11]: Are longer events processed more slowly because it takes more time to access the more diverse contexts associated with them, or because it takes more time to mentally re-enact the described events? The contrast we observe between time-to-perceive and semantic diversity points tentatively towards the latter account, at least for the concepts examined here.

We also controlled for visual perceptual strength [32] as a proxy for imageability (instead of controlling for concreteness ratings). The inclusion of this variable was motivated because (1) of work showing that participants’ performance on lexical tasks can be better predicted by perceptual strength than by concreteness [45], and because (2) visual perceptual strength is strongly related to imageability, and thus these models allow us to address the possibility that participants employed visual imagery when making judgments about time-to-perceive (e.g., visually imaging a *grapefruit*, or visualizing the events of Christmas day unfolding for *tradition*), and thus differences in how easy it is to visualize a concept, not time-to-perceive, account for the reported effects. However, the effect of time-to-perceive remained robust in these analyses too, indicating that the effect is not simply a byproduct of differences in imageability between, e.g., *grapefruit* and *tradition*.

Overall, our findings suggest that the time it takes to perceive something, independent of how spatially extended it is (e.g., because it contains many elements), how difficult it is to distinguish from other things, how ambiguous/semantically diverse it is, or how strongly imageable it is, affects how quickly we can conceive of it. Furthermore, the effect of time-to-perceive was present in three separate datasets and tasks. This consistency across tasks highlights the robustness of the finding and also suggests that information about time-to-perceive becomes available in tasks as shallow as lexical decision and word recognition, as well as in a more semantically demanding (concrete/abstract decision) task. Thus, our findings suggest that time-to-perceive is routinely simulated during conceptual retrieval, or, in other words, that time is an embodied property of concepts.

It is worth noting that by suggesting that during conceptual retrieval people simulate the (experience-based) information that they have acquired about *how long* it takes to perceive something, we do not mean to imply that the experiences from which the time-to-perceive information is derived are limited to direct sensory or motor experiences. For instance, a concept like *merit* is likely supported by apprehension of linguistic information that cues the designation of merit, and cultural information about how merit manifests in different contexts. We would contend that the amount of time it takes to integrate information from *any* source, be it sensory, motor, linguistic, etc., can affect how long it takes to perceive a given concept. In other words, there is no reason for the information that affects apprehension time to be restricted to sensory or motor information; it is likely that multiple sources of information contribute to how much time it takes to perceive a given concept (similar claims have been made regarding the information that shapes representations of event durations [12]).

In fact, this (source-neutral) feature of time-to-perceive means that it is a component of conceptual knowledge that can apply to concepts on the more abstract end of the spectrum, as well as to concepts that are more concrete. This is important because despite recent accounts emphasizing the role of language, emotion, and interoception in abstract concepts, they are still typically described in terms of what they are missing (e.g., “something you can’t see or touch”). We [20] and other researchers [18, 19] have hypothesized that one of the things that contributes to a concept being perceived as more abstract is that it requires integrating across more elements spread across time, and also that concepts are perceived as more abstract if they require more space to perceive. To test these hypotheses, we evaluated a linear model predicting concreteness ratings from our ratings of time, space, and (as a control) confusability. The model accounted for significant variance in concreteness ratings (*F*(3, 646) = 258.20, *p* < .001; *R*^2^ = .54), with both time (est = -0.34, *SE* = .07, *t* = -4.74, *p* < .001) and space (est = -0.67, *SE* = .05, *t* = -13.42, *p* < .001) as significant predictors: less concreteness (i.e., more abstractness) corresponded to higher ratings on both time- and space-to-perceive. Confusability was not a significant predictor in the model (est = -0.08, *SE* = .06, *t* = -1.37, *p* = .17), but given that confusability is strongly correlated with time-to-perceive (*r* = .85; in this model, the VIFs of confusability and time-to-perceive are 3.6 and 4.6, respectively, and that for space-to-perceive is 1.80) and that any variance that they share is not attributed to either individual predictor in the model, the true effect of both confusability and time-to-perceive may be underestimated. These results suggest that concepts that take more time and space to perceive are considered more abstract (as predicted by the framework developed in Davis et al. [20]), suggesting that the way the elements that constitute a concept are configured over time and space may be a contributing factor to what we think of as abstractness (for related work, see [16, 17, 19]).

While this is the first demonstration that certain temporal characteristics are re-instantiated when processing *concepts*, it builds on neuroscientific evidence that time is encoded in learning, and that a representation of time is maintained in long-term memory via the hippocampal system ([46]; for earlier demonstrations in rats, [47]; see also [48]). More directly related to the present work, our finding extends evidence that the temporal extension of *events* plays a role in language processing [12] to *concepts*.

### Limitations

Although we have shown that temporal properties are reactivated when people access conceptual knowledge, our findings leave open *which* aspect of time is reactivated. For instance, although we have focused on the time that it takes to *perceive* things, it is likely that for some things, “time-to-perceive” and “time-to-unfold” (or experience) are correlated—consider that both the time it takes to perceive that an *injection* is underway and the time it takes to experience an entire *injection* may be short, whereas both perceiving and experiencing *merit* may take longer. As described earlier, however, for many concepts in our dataset, particularly the more concrete ones (e.g., *dandelion*, *string*, *pepperoni*), there seems to be no sense in which they “unfold” as an experience (at least not beyond the construct of time-to-perceive). Because time-to-unfold is not a meaningful construct for these more concrete concepts, we can use them to assess whether the effect of time-to-perceive persists when there is unlikely to be a role for time-to-unfold.

To this end, we constrained our dataset to the 361 items with concreteness ratings over 4 (on a 1–5 scale) and conducted the same analyses that we conducted on the full dataset. The effect of time-to-perceive remained reliable in this subset of items, suggesting that at least for these items, time-to-perceive is part of conceptual knowledge. This is not to say that we have ruled out time-to-unfold as a component of conceptual knowledge. In fact, for concepts that *can* take time to play out (e.g., *effort*), we speculate that to the extent that both time-to-perceive and time-to-unfold have some degree of systematicity within concepts, they should both be part of conceptual knowledge. Future work is needed to develop a more fine-grained understanding of which and how temporal aspects of concepts are encoded in conceptual knowledge (for such work on events, see [12, 13]).

It is also important to acknowledge that a number of factors (in addition to clock duration) may contribute to our perception of how long something takes; for instance, for the time that it takes an *event* to unfold, this includes the number of subevents contained in the episode, and the complexity of those subevents ([13, 14]; for other factors, see [26–29]). For the focus of the current investigation—how long it takes to perceive the individual concepts that make up events—one might speculate that encoding, and, of most relevance here, simulation of time-to-perceive may be affected by the number and/or complexity of the features that must be apprehended. However, our analyses do not clearly support this conjecture. For instance, consider the amount of *space* that it takes to integrate the information necessary to perceive a concept. This measure is likely correlated with the number of elements being integrated, but although space-to-perceive was strongly correlated with time-to-perceive, it only predicted response times in one dataset (and only when the variance shared with time-to-perceive was not accounted for). Similarly, semantic diversity, which one also might imagine correlates with number of features, was only weakly correlated with time-to-perceive and was also not a significant predictor or response times.

The fact that we observed minimal, if any, influence of space-to-perceive or semantic diversity on response times suggests either that these measures are not good proxies for the number of features that must be apprehended, or that number of features does not play much of a role in the simulation of time-to-perceive. Future work should directly examine the relationship between the number and/or complexity of the features that must be apprehended and our encoding of time-to-perceive. In any case, our claim is not that clock duration *per se* (or some compressed function thereof) is directly simulated in language processing. Rather, the *perception* of how much time has elapsed is likely influenced by many factors, and it is this *perceived time* that we claim is reactivated when processing concepts in language.

Finally, there will always be additional lexical and/or semantic variables that could be included in the analyses. While we believe that we controlled for those most likely to be confounded with time-to-perceive (notwithstanding other time-related variables, which, if they contribute to the effects we observed, we could consider consistent with our approach), it is possible that relevant control variables were missed. In light of this, our data and scripts are available for exploration by other researchers.

## Conclusions

It is increasingly recognized that conceptual knowledge is experience-based [18, 49], and that a range of experiential knowledge is reactivated when we think about concepts (including abstract ones; for review, see [50]). Here, we broaden the scope of what is included in conceptual knowledge, demonstrating that one such experience is how long it takes for us to perceive an instance of concept: The longer it takes for us to perceive something in the world, the longer it takes to play out in our minds.

## Supporting information

S1 File Reproducible scripts and data files. (DOCX)

S1 Appendix Instructions for space-to-perceive rating task. (DOCX)

S2 Appendix Instructions for confusability rating task. (DOCX)

S1 Table Model results for response times in word recognition, lexical decision, and semantic decision tasks with semantic diversity as an additional control. (DOCX)

S2 Table Model results for response times in word recognition, lexical decision, and semantic decision tasks with imageability as an additional control. (DOCX)
