# Supplementary material for: Is time an embodied property of concepts?
Source: PLoS One. 2023 Sep 5;18(9):e0290997. doi: 10.1371/journal.pone.0290997 (PMC10479924; doi:10.1371/journal.pone.0290997)
Supplement: S1 File — (DOCX) [file pone.0290997.s001.docx]

**S1 Supporting Information. Reproducible Scripts and Data Files.**

All other exploratory analyses, as well as scripts and data to reproduce all reported analyses are available on the Open Science Framework (<https://osf.io/q2gdt/>)
