# Supplementary material for: Is time an embodied property of concepts?
Source: PLoS One. 2023 Sep 5;18(9):e0290997. doi: 10.1371/journal.pone.0290997 (PMC10479924; doi:10.1371/journal.pone.0290997)
Supplement: S1 Appendix — (DOCX) [file pone.0290997.s002.docx]

**S1 Appendix. Instructions for Space-to-Perceive Rating Task.**

For measuring the *space* dimension, the following instructions were used, where participants rated each item from 1 (*very little space*) to 7 (*a lot of space*).

*You will be asked how much space one needs to accumulate the information necessary to perceive different things. 

For example, some things, like a bowl, can be perceived in a small area—e.g., its shape and size can be perceived by looking in a relatively small space. But perceiving other things, like tradition, may require attention to multiple events spread across many places, or a large area. Still other things, like gamble or thinking may lie somewhere in between. 

Make your responses based on how much space it would require to accumulate the information necessary to perceive the parts that make up each thing. 

We are****not****interested in how easy it is to tell each thing apart from something similar. For example, even though it may be difficult to distinguish a banjo from, e.g., a mandolin, it does not take much space to perceive the parts that make up a banjo/mandolin-like thing. A banjo would therefore likely receive a response on the “very little space” end of the scale. 

We are also****not****interested in how familiar you are with each thing. For example, even if you are more familiar with banjos than with mandolins, they share similar properties and therefore it takes about the same amount of space to perceive the parts that make up a banjo/mandolin-like thing. Both a banjo and a mandolin would therefore likely receive a response on the “very little space” end of the scale.

Please tell us how much space you think it would take to accumulate the information necessary to perceive or recognize the following things. There are no right answers, so simply go with your first instinct.*
