# Supplementary material for: Is time an embodied property of concepts?
Source: PLoS One. 2023 Sep 5;18(9):e0290997. doi: 10.1371/journal.pone.0290997 (PMC10479924; doi:10.1371/journal.pone.0290997)
Supplement: S2 Appendix — (DOCX) [file pone.0290997.s003.docx]

**S2 Appendix. Instructions for Confusability Rating Task.**

For measuring the *confusability* dimension, participants were asked to rate each item from 1 (*very easy*) to 7 (*very difficult*) given the following instructions:

*You will be asked how difficult it is to distinguish something from similar things. 

For example, a bowl has a characteristic shape and size that makes it easily recognizable, and distinct from other objects. But other things, like tradition, may be more difficult to recognize. It may be difficult to distinguish between whether you are observing a tradition or a one-time event.

Make your responses based on how difficult it would be to distinguish each thing from similar things.

We are****not****interested in how much time it would take to perceive each thing. For example, even though you may be able to quickly perceive the parts of a banjo, a banjo could still receive a score on the “more difficult” end of the scale. This is because a banjo and a mandolin share many physical properties, meaning that it may be hard to distinguish them from one another. 

We are also****not****interested in how familiar you are with each thing. For example, even if you are more familiar with banjos than with mandolins, it is difficult to distinguish a banjo from a mandolin. Both a banjo and a mandolin would therefore likely receive a response on the “more difficult” end of the scale.

Please tell us how difficult you think it would be to distinguish each thing from similar things. There are no right answers, so simply go with your first instinct*
