# Supplementary material for: Is time an embodied property of concepts?
Source: PLoS One. 2023 Sep 5;18(9):e0290997. doi: 10.1371/journal.pone.0290997 (PMC10479924; doi:10.1371/journal.pone.0290997)
Supplement: S1 Table — (DOCX) [file pone.0290997.s004.docx]

**S1 Table. Model Results for Response Times in Word Recognition, Lexical Decision, and Semantic Decision Tasks with Semantic Diversity as an Additional Control.**

|  | Lexical Decision  (ELP; Balota et al., 2007)  *N*_words_ = 459 | | | | Word Recognition  (ECP; Mandera et al., 2019)  *N*_words_ = 469 | | | | | Semantic Decision  (SDP; Pexman et al., 2017)  *N*_words_ = 467 | | | | |
| --- | --- | --- | --- | --- | --- | --- | --- | --- | --- | --- | --- | --- | --- | --- |
| **Model 1** | **est** | ***SE*** | ***t*** | ***P*** | **est** | ***SE*** | ***t*** | ***p*** | | | **est** | ***SE*** | ***t*** | ***p*** |
| Length | 12.28 | 1.66 | 7.37 | **< .001** | 10.66 | 1.88 | 5.66 | | **< .001** | | 6.87 | 2.67 | 2.57 | **.010** |
| Frequency | -21.00 | 2.94 | -7.15 | **< .001** | -20.10 | 3.31 | -6.08 | | **< .001** | | -5.50 | 4.69 | -1.17 | .24 |
| AoA | 12.02 | 1.69 | 7.10 | **< .001** | 14.12 | 1.91 | 7.41 | | **< .001** | | 12.98 | 2.74 | 4.74 | **< .001** |
| Concreteness | 1.06 | 3.61 | 0.29 | .77 | 8.05 | 4.08 | 1.97 | | **.049** | | -10.04 | 6.40 | -1.57 | .12 |
| Distance*  (to concreteness midpoint) | -- | -- | -- | -- | -- | -- | -- | | -- | | -128.53 | 15.43 | -8.33 | **< .001** |
| SemD | -6.06 | 12.15 | -0.50 | .62 | -12.88 | 13.67 | -0.94 | | .35 | | -12.41 | 19.25 | -0.64 | .52 |
| **Total variance explained** | *R*^2^ = .48 | | | | *R*^2^ = .43 | | | | | | *R*^2^ = .40 | | | |
| **Model 2** |  | | | | | | | | | | | | | |
| Length | 11.57 | 1.76 | 6.58 | **< .001** | 10.94 | 1.95 | 5.60 | | **< .001** | | 7.39 | 2.83 | 2.62 | **.009** |
| Frequency | -22.94 | 3.11 | -7.38 | **< .001** | -21.33 | 3.44 | -6.20 | | **< .001** | | -4.32 | 4.98 | -0.87 | .37 |
| AoA | 9.52 | 1.98 | 4.80 | **< .001** | 8.57 | 2.20 | 3.90 | | **< .001** | | 13.98 | 3.17 | 4.40 | **< .001** |
| Concreteness | 8.65 | 4.70 | 1.84 | .07 | 19.35 | 5.19 | 3.73 | | **< .001** | | -13.60 | 7.75 | -1.75 | .08 |
| Distance*  (to concreteness midpoint) | -- | -- | -- | -- | -- | -- | -- | | -- | | -130.81 | 15.72 | -8.32 | **< .001** |
| SemD | -2.73 | 12.17 | -0.22 | .82 | -9.61 | 13.44 | -0.72 | | .47 | | -14.02 | 19.38 | -0.72 | .47 |
| Confusability | -8.04 | 5.32 | -1.51 | .13 | 26.89 | 5.88 | 4.58 | | **< .001** | | -2.29 | 8.51 | -0.27 | .79 |
| Space | 11.50 | 6.42 | 1.79 | .07 | 6.75 | 7.13 | 0.95 | | .34 | | -7.53 | 10.38 | -0.73 | .47 |
| **Total variance explained** | *R*^2^ = .49, ∆*R*^2^ = .01  *F*(2, 451) = 3.28, *p* = .04 | | | | *R*^2^ = .46, ∆*R*^2^ = .03 *F*(2, 461) = 11.93, *p* < .001 | | | | | | *R*^2^ = .40, ∆*R*^2^ = .00  *F*(2, 458) = 0.34, *p* = .72 | | | |
| **Model 3** |  | | | | | | | | | | | | | |
| Length | 12.28 | 1.74 | 7.07 | **< .001** | 11.54 | 1.95 | 5.93 | | **< .001** | | 8.07 | 2.83 | 2.85 | **.005** |
| Frequency | -20.46 | 3.11 | -6.57 | **< .001** | -19.41 | 3.47 | -5.60 | | **< .001** | | -2.18 | 5.04 | -0.43 | .67 |
| AoA | 7.56 | 2.01 | 3.76 | **< .001** | 6.92 | 2.25 | 3.08 | | **.002** | | 12.05 | 3.27 | 3.69 | **< .001** |
| Concreteness | 11.90 | 4.68 | 2.54 | **.01** | 22.02 | 5.22 | 4.22 | | **< .001** | | -9.97 | 7.88 | -1.26 | .21 |
| Distance*  (to concreteness midpoint) | -- | -- | -- | -- | -- | -- | -- | | -- | | -135.04 | 15.75 | -8.57 | **< .001** |
| SemD | -6.55 | 12.00 | -0.55 | .59 | -12.74 | 13.37 | -0.95 | | .34 | | -17.40 | 19.35 | -0.90 | .37 |
| Confusability | -9.01 | 6.67 | -1.35 | .18 | 13.15 | 7.43 | 1.77 | | .08 | | -17.97 | 10.89 | -1.65 | .10 |
| Space | 1.93 | 6.72 | 0.29 | .77 | -0.97 | 7.53 | -0.13 | | .90 | | -16.47 | 11.04 | -1.49 | .14 |
| Time | 36.66 | 8.91 | 4.12 | **< .001** | 29.69 | 9.96 | 2.98 | | **.003** | | 33.34 | 14.54 | 2.29 | **.022** |
| **Total variance explained** | *R*^2^ = .51, ∆*R*^2^ = .02  *F*(1, 450) = 16.93, *p* < .001 | | | | *R*^2^ = .47, ∆*R*^2^ = .01 *F*(1, 460) = 8.89, *p* = .003 | | | | | | *R*^2^ = .41, ∆*R*^2^ = .01  *F*(1, 457) = 5.25, *p* = .022 | | | |

*Note*. ECP = English Crowdsourcing Project; ELP = English Lexicon Project; SDP = Calgary Semantic Decision Project; AoA = Age of Acquisition; SemD = Semantic Diversity. The summary in the final row shows the results of a model comparison of Model 2 to Model 1. This was included (only in the semantic decision models) because judging whether a word is concrete or abstract is presumably most difficult near the midpoint of the scale (i.e., in boundary cases), and so accounting for distance allows us to better capture variation in semantic decision RTs. (Without it, the patterns were the same, and time-to-perceive was still a reliable predictor of response times, but the total variance explained by the semantic decision models was substantially lower: *R^2^* = ~.30.)
